# Evaluation and Management of Failed Shoulder Instability Surgery

**DOI:** 10.2174/1874325001711010897

**Published:** 2017-08-31

**Authors:** António Cartucho, Nuno Moura, Marco Sarmento

**Affiliations:** Orthopaedic Department Cuf Descobertas Hospital Rua Mário Botas 1998-018 Lisbon – Portugal

**Keywords:** Failed shoulder instability surgery, Arthroscopy, Bankart, Latarjet, Remplissage, Tricortical iliac graft, Bone defect

## Abstract

**Background:**

Failed shoulder instability surgery is mostly considered to be the recurrence of shoulder dislocation but subluxation, painful or non-reliable shoulder are also reasons for patient dissatisfaction and should be considered in the notion.

**Methods:**

The authors performed a revision of the literature and online contents on evaluation and management of failed shoulder instability surgery.

**Results:**

When we look at the reasons for failure of shoulder instability surgery we point the finger at poor patient selection, technical error and an additional traumatic event. More than 80% of surgical failures, for shoulder instability, are associated with bone loss. Quantification of glenoid bone loss and investigation of an engaging Hill-Sachs lesion are determining facts. Adequate imaging studies are determinant to assess labrum and capsular lesions and to rule out associated pathology as rotator cuff tears. CT-scan is the method of choice to diagnose and quantify bone loss. Arthroscopic soft tissue procedures are indicated in patients with minimal bone loss and no contact sports. Open soft tissue procedures should be performed in patients with small bone defects, with hiperlaxity and practicing contact sports. Soft tissue techniques, as postero-inferior capsular plication and remplissage, may be used in patients with less than 25% of glenoid bone loss and Hill-Sachs lesions. Bone block procedures should be used for glenoid larger bone defects in the presence of an engaging Hill-Sachs lesion or in the presence of poor soft tissue quality. A tricortical iliac crest graft may be used as a primary procedure or as a salvage procedure after failure of a Bristow or a Latarjet procedure. Less frequently, the surgeon has to address the Hill-Sachs lesion. When a 30% loss of humeral head circumference is present a filling graft should be used.

**Conclusion:**

Reasons for failure are multifactorial. In order to address this entity, surgeons must correctly identify the causes and tailor the right solution.

## 1 INTRODUCTION

Shoulder instability is one of the most frequent clinical entities in sports traumatology. Due to basic science and clinical investigation advances, there has been a constant evolution in concepts. Conservative treatment is indicated in atraumatic instability. Surgical treatment should be considered for first time dislocation in some group of patients and has formal indication on the recurrent traumatic unidirectional instability [1]. Anatomic reconstruction with a non-aggressive technique is the goal of primary surgical treatment, nevertheless due to numerous factors, failure rates after primary open or arthroscopic surgery are noted to be between 5% and 30% [2-4].

In order to analyse and manage failure, the concept itself, has to be defined. The most obvious indication for surgery is recurrence of dislocation but sub-luxation, painful or non-reliable shoulder are also reasons for patient dissatisfaction and should also be considered in the notion.

After defining the type of clinical failure, the surgeon should try to understand the underlying causes, in order to use them at the time of the revision surgery.

## 2 REASONS FOR FAILURE

There has been an ongoing argument about failure rate after shoulder instability treatment performed open versus arthroscopically. In a recent systematic review [5] the rate of recurrent instability after arthroscopic procedures was not statistically different from the recurrence rate after open procedures. It is not the technique, that leads to failure but what one is capable of doing with it.

Failure of shoulder instability surgery may be due to poor patient selection, technical error and an additional traumatic incident.

### 2.1 Patient Selection

#### 2.1.1 Clinical Evaluation

From a clinical point of view; age, and gender, have been pointed at, as risk factors for recurrence of instability after a primary shoulder dislocation [6]. The recurrence rate of instability after surgery was associated with patients less than 22 years of age at the time of first dislocation, male in gender and with more than six months between the first dislocation and time of surgery [7].

Clinical evaluation should quantify hiperlaxity according to Beighton [8], search for major instability signs like apprehension and relocation tests but also for minor instability signs like O’Brien [9], biceps load [10] and hiperabduction test of Olivier Gagey [11]. Loss of motion might be due to overtightening of the capsulolabral complex or due to secondary complications as hardware impingement or chondrolysis. The rotator cuff function should be evaluated, especially the subscapularis, using the bear-hug [12] and press belly tests. The lift-off and the belly–press tests may be influenced by restricted internal rotation.

Apart from instability signs, associated lesions such as fractures, rotator cuff tears and neurologic lesions should be suspected as they interfere with the treatment strategy and timing.

Rotator cuff involvement after traumatic dislocation of the shoulder has a high incidence in patients over 40 years of age with an incidence in the current literature ranging from 35% to 86% [13-15]. Clinical suspicion is based on loss of active motion and strength of the affected tendons. Differential diagnosis should be made from neurologic lesions. The lack of altered sensibility and an isolated loss of active power of a tendon are indicators of a tendinous lesion. In these cases a Magnetic Resonance Imaging (MRI) should be performed in order to assess the type and the location of the rupture.

Axillary nerve palsy is a common associated lesion, with an incidence of 48% across all age groups [16]. Lesions of the suprascapular nerve have also been documented but in a much smaller scale. Risk factors associated with nerve lesions are age, (increasing risk factor 1.3 times for every 10 year period), haematoma, (four times greater risk with significant bruising of the shoulder) and associated fractures (double the risk factor). For axillar nerve palsy the main clinical signs are an altered sensibility of the deltoid region and paresis of the deltoid with loss of active abduction.

#### 2.1.2 Imaging Evaluation

**Fig. (1):**
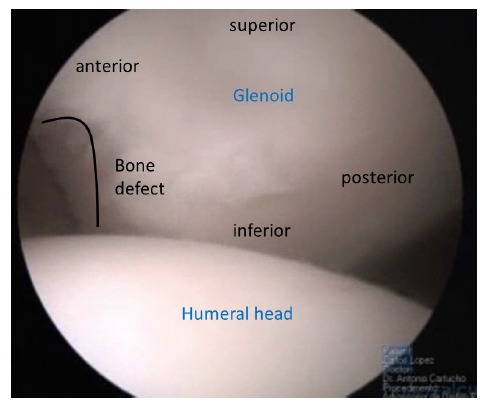
Inverted peer.

More than 80% of surgical failures for shoulder instability are associated with bone loss [2]. Since the paper from Itoi [17] and all, we know that a small loss of anterior glenoid bone rim (6 to 8mm) equal to 21% of the glenoid surface, is the limit for a successful Bankart procedure. With glenoid bony defects greater than 21% the shoulder showed persistent instability. For those patients with significant anterior glenoid bone loss, Burkhart and de Beer, developed the concept of “inverted pear glenoid” (Fig. **1**) as the one having a smaller inferior than superior diameter [2]. This type of defect can be due to chronic bone loss caused by repetitive anterior glenoid rim erosion or an acute bone fracture as described by Sugaya [18]. Besides glenoid bone loss, humeral head bone loss, the Hill-Sachs lesion, is a major factor of shoulder instability. The engaging Hill-Sachs concept by Burkhart *et al.* [2] refers to a defect “that presents its long axis parallel to anterior glenoid with the shoulder in a functional position of abduction and external rotation, so the Hill-Sachs lesion engages the corner of the glenoid”. Every Hill-Sachs lesion may eventually engage. The fact is that the great majority will engage in non-functional positions of the shoulder and do not contribute to shoulder instability as much as the ones that engage in functional positions. Apart from the position and the axis of the Hill-Sachs lesion the percentage of articular surface involved is also a concern with authors describing a major contribution of this fact for recurrent instability [19]. The association of glenoid and humeral bone loss raised the concept of glenoid tracking [20]. When there is no glenoid defect the glenoid track is 84% +/- 14% of the glenoid width. When there is a bony defect, its width should be subtracted from the 84% length of the glenoid track, subsequently obtaining the correct measurement. If the medial margin of the Hill- Sachs lesion is more medial than the glenoid track soft tissue procedures are unlikely to restore shoulder stability.

**Fig. (2):**
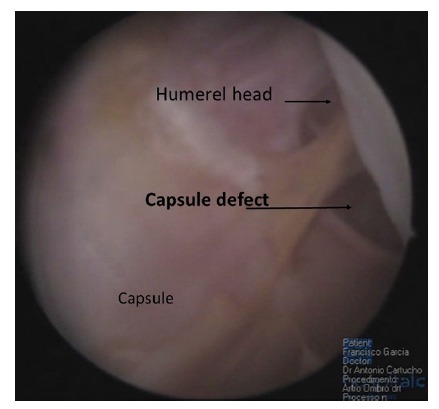
HAGL lesion.

Soft tissue quality, capsular distention, superior labrum anterior and posterior (SLAP) lesions, anterior labral periosteal sleeve avulsion (ALPSA) lesions, humeral avulsion glenohumeral ligament (HAGL) lesion (Fig. **2**), and concomitant cuff lesions have also been associated with greater incidence of failure in shoulder surgery for instability [21].

Adequate imaging studies are of upmost importance to assess bone loss, soft tissue type of lesions and tissue quality. AP view and axillary radiographs such as the West Point view may show bone loss. CT- Scan should be performed in patients with bone lesions in plain radiographs and in those with multiple dislocations and apprehension at lower abduction angles on examination [22]. Axial and sagittal views permit visualization of glenoid and humeral head defects. Several methods have been described to quantify bone loss namely using three-dimensionally reconstructed CT images with subtraction of the humeral head [18]. As the inferior glenoid has consistently shown to be a “circle” it is possible to determine the percentage of bone loss trough determination of the area of the circle and the area of the glenoid bone defect [23]. Three-dimensional CT scan can also be used to determine the length of the glenoid lesion. Gerber *et al.* describe that if the length of the lesion was equal to half the widest diameter of the glenoid in the AP plane the resistance to dislocation was decreased by 30% [24].

Artro MRI is the method of choice to evaluate and classify labrum lesions, concomitant rotator cuff tears and capsular laxity with an accuracy of 91, 9% in assessing soft tissue pathology in patients with recurrent instability when compared to arthroscopic findings [7, 25, 26].

### 2.2 Technical Errors

Technical errors can be divided into failure of correct assessment of factors contributing to instability and surgical errors.

As said previously, surgical failure for shoulder instability is associated with bone loss. Failure to correctly identify and quantify the glenoid and/or humeral head bone loss is associated with higher recurrence rates mainly in patients who participate in contact sports. Also the choice of the bone block procedure can influence the recurrence rate. Bristow procedure is associated with a greater recurrence rate than the Latarjet [27] procedure. Technical failure might be due to incorrect position of the bone fragment, resorption or fracture of the bone fragment and “hardware” complications like screw breakage or joint penetration.

Errors associated with soft tissue management may also come from misdiagnosis especially for posterior labral lesions and humeral avulsion glenohumeral ligament (HAGL) lesions. A careful inspection of superior labrum is also important in order to comprehend its contribution to instability. Due to the great number of normal variants of this labrum region, the most common error consists in overtreatment, leading to stiffness and secondary pathology of long head of the biceps. Surgical errors, when addressing soft tissue, consist in insufficient mobilization, insufficient capsular plication and superior transfer. Poor positioning of the suture anchor, either too medial (leads to instability) or too proud (produces a degenerated joint in a short period of time), must be avoid.

### 2.3 New Traumatic Episode

The type of incident associated with a recurrence, may be indicative of the degree of shoulder instability. If a simple gesture of daily living activity produces the re-dislocation, most likely an engaging Hill-Sachs lesion is present [2]. On the other hand, a high energy trauma may produce new lesions that have to be addressed.

## 3 TREATMENT OPTIONS

### 3.1 Conservative Treatment and Associated Lesions Management

After a recurrence, the first treatment attitudes should promote comfort namely a sling for support. Pain killers and anti-inflammatory should be prescribed. A search for possible associated lesions like axillary nerve palsy, rotator cuff rupture and great tuberosity fractures should be performed.

Conservative treatment for instability recurrence after shoulder instability surgery should be considered only for patients with low functional demands and no associated complications [28]. This is due to the fact that results are quite discrepant regarding the evaluation scores and consequent clinical results.

Associated lesions as rotator cuff tears and axillary nerve palsy must be considered in the treatment algorithm. If a rotator cuff tear is diagnosed, treatment options vary according to the age of the patient, the location and type of rupture. A complete acute rupture before the age of 40 years is an indication for surgery. On the other hand, patients older than 40 with a partial rupture or an acute on chronic rupture with a grade two retraction and atrophy should be managed conservatively. Surgical indication should only take place in case of failure of these measures [13-15]. If a neurologic lesion is diagnosed, conservative treatment should be considered due to the fact that more than 95% of the neurologic lesions are reversible. This will consists in an assiduous physiotherapy programme to maintain passive range of motion in order to avoid stiffness . If three weeks after the episode of dislocation there is no significant improvement of the neurologic status, an electromyogram should be performed in order to search for re-enervation signs and to confirm the diagnosis. If after three months the palsy persists a surgical exploration is indicated [16].

### 3.2 Surgical Treatment

Surgical treatment is the gold standard when dealing with recurrence after shoulder instability surgery but the type of procedure and the technical tools to apply in each case must be carefully decided in order to avoid more than one surgery after the initial failure. This fact is of upmost importance because we know from the literature that the clinical results of patients with one reoperation are higher than the ones with more than one reintervention [28]. Open repair techniques for failed anterior shoulder instability have been reported in several studies to have lower recurrence rates than arthroscopic techniques [28, 30]. Nevertheless in more recent studies using arthroscopic procedures, the incidence of recurrence was similar for both techniques [31-33]. The recurrence rate after revision surgery was reported by Levine *et al.* as 22%, more than double than primary repair [34].

The choice of the technique should take into account the surgeons experience, the age and activity of the patient, the type of ligamentous lesion, the capsule-labral quality and last but not least, the presence of a glenoid bone loss greater than 20% and/or the existence of an engaging Hill-Sachs lesion.

#### 3.2.1 Arthroscopic Capsule-Labral Reconstruction Procedures

Arthroscopic procedures have several advantages: they allow assessing problems in the capsule-labral complex visualizing a 360º angle circumference, they produce less scar tissue, reduce postoperative pain, reduce sub scapularis iatrogenic damage and lead to less restriction of passive range motion a fact that can be important for overhead athletes. Patients with good capsular tissue, minimal bone loss, with an evaluation error or with an identified technical error at the first operation and with a recurrent significant trauma are good candidates for this revision technique. It is important not only to anatomically reduce de labrum but also to promote an inferior and postero-inferior capsular plication to eliminate the redundant inferior capsular pouch [32]. Remaining sutures and anchors from previous surgeries should be extracted if they interfere with the capsule-labral reconstruction or healing and if the joint integrity is at risk. Polyetheretherketone (PEEK) anchors can be over drilled and substituted by broader anchors.

Arthroscopy may be performed on lateral decubitus or on beach-chair position and standard posterior and antero-superior and inferior portals are used. Confirmation of minimal bone loss and non-existence of engaging Hill-Sachs lesion may be confirmed during the procedure using the methods described by Burkhart *et al.* [2] and a 360º inspection of the capsule-labral complex should be performed. The anterior labrum must be mobilized and if a bone fragment is present, its attachment to the labrum should be preserved, in order to integrate it in the anterior reconstruction. Inspection of the postero-inferior capsule-labral complex is an important step. At the time of dislocation, this region is also stressed and labral tears, capsular elongation, or Kim’s type lesions can be produced [33]. These lesions have to be addressed and with the arthroscope in the antero-superior portal, using the posterior and antero-inferior portals as working portals, a postero inferior labral lesion can be mobilized and repaired with suture anchors placed through a postero-superior accessory portal Fig. (**3**). In this process, the surgeon may choose the amount of capsular plication needed. As the main instability is in the anterior direction it is advisable to tie this posterior knots after the anterior repair is finished. In order to decrease capsular volume, an interval closure technique has been proposed if this space was considered to be redundant or if the patient still had a sulcus sign in external rotation [32].

When surgical indication is correct, good or excellent results should be expected in around 83% of the cases, with minimal loss of external rotation at the side and a return to pre injury level of activity. Kim *et al.* in revision of 23 patients addressed arthroscopically with a Bankart procedure, capsular plication and shift, for a failed instability repair presented good and excellent results in 83% of the study population, having a total of 78% returning to previous activities but a recurrence rate of 22% [33]. Creighton *et al.* present a series of eighteen patients with failed traumatic instability repairs treated with revision arthroscopic labral fixation and plication with a mean follow-up of 29.7 months. Glenoid bone loss greater than 25% was considered an exclusion criteria. All patients had recurrent anterior-inferior labral tears at the time of surgery. On the basis of a circle concept with a 360°, the mean degree of labral/capsular injury was 155°. Labral repair included, on average, 4 suture anchors and 3 plication stitches, and 15 of the 18 patients received a rotator interval closure. There was significant improvement of pain scores: Simple Shoulder Test and American Shoulder and Elbow Surgeons scores. The authors conclude that the results are satisfactory in selected patients [32].

#### 3.2.2 Open Capsule-Labral Reconstruction Procedures

Patient specific and pathoanantomic factors are at the base of decisions for this procedure. Collision athletes, patients with generalized hyper laxity according to Beighton criteria, bone loss greater than 15% and less than 20% of the glenoid surface, the presence of an ALPSA or an HAGL lesion and poor capsule-labral tissue are associated with poorer prognosis [21]. Relative indications for open procedure are exposed hardware and subscapularis deficiency [35].

The procedure is performed by a deltopectoral approach, between the coracoid/conjoint tendon and the long head of the biceps, superior to the “three sisters” a subscapularis split should be performed separating this muscle from the capsule. An incision should be made on the capsule reaching the labrum insertion on the anterior glenoid defect. Mobilization of the labrum should be performed and identification and eventual removal of previous hardware should be done. After careful preparation of the bone, the anterior glenoid rim and capsular redundancy evaluation, reinsertion of the labrum with suture anchors and antero-inferior capsular shift should be performed.

As seen previously the results of the revision procedure are inferior to the primary stabilization. In a group of 50 patients, with previous failed stabilization, treated with an open Bankart procedure and a capsular shift, Levine *et al.* [34] presents 78% of good or excellent results with similar results only present in 67% of the atraumatic instability patients at a mean follow up of 4,7 years. The authors considered, atraumatic and voluntary instability as well as multiple stabilization procedures to be risk factors for a poorer outcome. Cho *et al*. [36] present a study on twenty-six shoulders that performed traditional open Bankart repair as revision surgery after a failed arthroscopic Bankart procedure for traumatic anterior shoulder instability. With a mean follow up of 42 months 88, 5% of the patients had good clinical results with a mean Rowe score of 81. A statistically significant difference on pre and post-operative flexion and abduction from 173º to 164º of flexion and from 65º to 55º of external rotation was described. Recurrence of instability was present in 11.5% of the patients. The authors associate this poor results to the presence of engaging Hill-Sachs lesion and shoulder hiperlaxity.

Zabinski *et al.* [37] in their study of 43 patients who underwent shoulder stabilization also describe a significant difference of 78% to 39% of good results from traumatic versus atraumatic shoulder instability patients.

#### 3.2.3 Latarjet and Other Bone Block Procedures for Anterior Glenoid Defects

The percentage of patients with recurrent dislocation, after instability surgery, that have evidence of bone defect is reported to be as high as 73% to 86% as opposed to the patients with acute dislocation that have a reported incidence between 22% and 41% [2, 38].

Relevant bone loss on the glenoid surface, especially when associated with a Hill-Sachs lesion, significantly reduces stability. Bone procedures are necessary most of the time to address this type of patients.

**Fig. (4):**
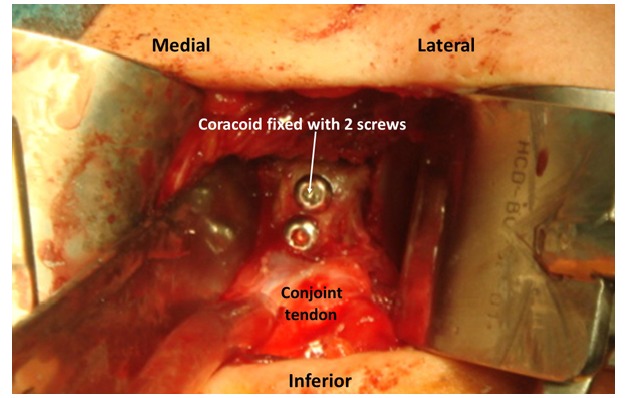
Latarjet.

The most common bone replacement technique is the transfer of the coracoid to the anterior glenoid rim. Two major techniques are used, the Bristow [39] procedure with the transfer of the tip of the coracoid and the conjoint tendon that is fixed upright on the anterior glenoid defect with a screw and the Latarjet [27] procedure, that transfers the coracoid from the tip to the neck with the conjoint tendon. The coracoid is fixed with two screws to the glenoid anterior defect by its inferior surface (Fig. **4**). Both procedures are performed trough a deltopectoral approach and the subscapularis should be incised with a longitudinal split approach, exposing the anterior capsule. Correct positioning of the coracoid in the anterior glenoid rim is crucial. When proximal or medial, residual instability may be maintained, but, when lateral or “proud” into the joint, the incidence of early arthrosis is higher [40]. Apart from these complications, coracoid pseudarthrosis and hardware problems have also been reported. In a recent paper, Schmidt *et al.* [41] present the results of 49 patients with previous failed stabilizations treated with a Latarjet procedure. No revision surgery was needed. Forty-three shoulders (88%) were subjectively graded as excellent or good; three, fair; and three, poor. The mean subjective shoulder value increased from 53% preoperatively to 79% at the time of follow-up, and the Constant-Murley score remained high. Optimal graft placement was obtained in thirty cases and was related to better clinical outcome and less progression of osteoarthritis than was suboptimal graft placement. The authors conclude that this technique restores shoulder stability but patients with pre-operative pain may be dissatisfied due to its persistence.

More recently arthroscopic techniques for Bristow and Latarjet procedures have been described [42, 43] with good preliminary results. Lafosse *et al.* [44] report the series of the first 100 patients treated with an arthroscopic Latarjet with a mean follow up of 26 months. Patient-reported outcomes revealed 91% excellent scores and 9% good. Range of motion showed an average loss of external rotation of 18º. Perioperative complications (4%) included 2 hematomas, 1 graft fracture, and 1 transient musculocutaneous nerve palsy. Late complications included 4 cases of graft non-union and 3 of graft lysis. In this series, graft positioning was not completely correct in 20% of the cases. There are no published data concerning the use of these techniques after failed instability reconstruction.

Glenoid reconstructions may also be achieved with iliac crest bone graft. In Warner’s [45] paper with a mean follow-up of 33 months, the mean American Shoulder and Elbow Surgeons score was 94, compared with a preoperative score of 65. The University of California, Los Angeles score improved from 18 to 33. The Rowe score improved from a preoperative score of 28 to 94. The mean motion loss compared with the contralateral, normal shoulder was 7 degrees of flexion, 14 degrees of external rotation in abduction, and one spinous process level for internal rotation. There were no recurrences, with all patients returning to pre injury activity levels. Control CT-scans demonstrated osteo-integration of the graft and no signs of arthritic changes at 33 months of follow-up. More recently arthroscopic techniques have been described using tricortical iliac crest bone graft which is passed through a 10mm antero-inferior arthroscopic cannula and guided to the anterior glenoid bone defect being subsequently fixed with two screws. The capsule and remaining labrum is then fixed to the graft with suture anchors. Nevertheless there are no published results of this approach [46]. In another paper, using a tricortical autogenous graft, Kraus *et al.* [47] described an arthroscopic delivery and fixation technique, presenting good or excellent results and without major complication after a mean follow up of 20 months.

#### 3.2.4 Capsulodesis and Other Techniques to Address Hill-Sachs Lesions

**Fig. (5):**
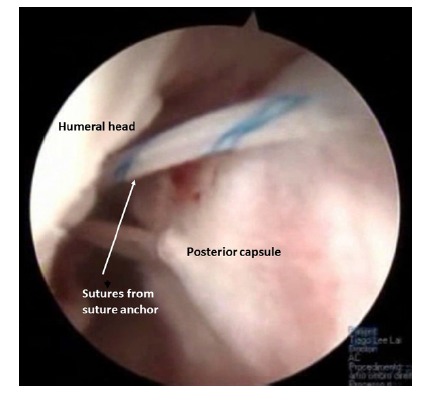
Remplissage.

Recently an arthroscopic posterior capsulodesis technique has been described for engaging Hill-Sachs lesions. After preparation of the Hill-Sachs lesion one or two suture anchors are placed and the posterior capsule and infraspinatus are pierced preparing the filling of the Hill-Sachs (Fig. **5**). The knots should only be tied after reconstruction of the anterior capsulo-labral lesions [48]. In a recent paper Wolf *et al.* [49] analysed 59 patients with glenoid bone loss <25% that received an arthroscopic remplissage associated to a Bankart procedure with a mean follow up of 58 months. The mean Rowe score was 95, the Constant score also classified as 95 and Western Ontario Shoulder Instability Index at 110. All patients, except for 2 traumatic dislocations (4. 4%), had no reoperations or complications.

Osseous allograft reconstruction exists as a solution to address moderate to large humeral-sided defects (>40% articular surface) in younger patients. Several authors [50-52] have published papers on the role of this procedure in the setting of chronic anterior instability with associated bone loss. This procedure attempts to fill larger defects with both a structural and osteoconductive material in an attempt to avoid prosthetic replacement. Specific indications mainly restrict this procedure to younger patients with larger sized defects that do not have a significant degree of osteopenia or degenerative joint disease. After an appropriate preoperative evaluation by CT scan to quantify humeral head defect, a sized matched fresh-frozen humeral or femoral head is obtained and used to graft the defect. The authors used a deltopectoral approach and a sized matched allograft measuring 2 mm wider than the actual defect is impacted and secured with two cancellous screws placed through the anterolateral humeral cortex. This decreases the risk of hardware prominence after partial resorption.

Patients with a humeral head deficiency greater than 30% were addressed by Miniaci *et al* [53] with bone grafting of the bone defect with a tailored allograft in conjunction to anterior capsulo-labral reconstruction. Eighteen patients with an average age of 31.5 (18–52) were reviewed at a medium period of fifty months (24–96). All patients had resolved their shoulder instability with no documented recurrences. All patients had severe apprehension in the pre-operative period a problem that was resolved completely in fifteen patients. Average loss of external rotation was measured pre-operatively at forty degrees and improved to ten degrees in the postoperative period. Two patients had partial collapse of the graft with symptoms of pain requiring screw removal. Patients had an average Constant score of eighty-seven postop. Subjectively all patients would repeat the procedure.

## CONCLUSION

Treatment of failed shoulder instability surgery is mainly surgical and depends on the reasons for recurrence. Patient clinical evaluation and imaging are of upmost importance to determine the correct procedure. Surgical treatment options must take into account bone loss, soft tissue quality and specific patient factors. Small bone loss can be addressed with an anterior capsule-labral reconstruction and complementary techniques such as remplissage. If major bone loss is present, bone block procedures are indicated. Patients presented with a Hill-Sachs lesion greater than 30% may need a filling graft. Results of revision surgery are worse than primary surgery and degrades if further surgeries are necessary, turning it absolutely determinant, to get it wright at the first reintervention.

## Figures and Tables

**Fig. (3):**
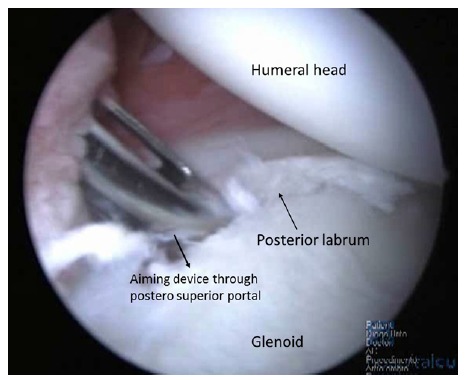
Postero superior portal.

## References

[1] Kirkley A., Werstine R., Ratjek A., Griffin S. (2005). Prospective randomized clinical trial comparing the effectiveness of immediate arthroscopic stabilization versus immobilization and rehabilitation in first traumatic anterior dislocations of the shoulder: Long-term evaluation.. Arthroscopy.

[2] Burkhart S.S., De Beer J.F. (2000). Traumatic glenohumeral bone defects and their relationship to failure of arthroscopic Bankart repairs: significance of the inverted-pear glenoid and the humeral engaging Hill-Sachs lesion.. Arthroscopy.

[3] Barnes C.J., Getelman M.H., Snyder S.J. (2009). Results of arthroscopic revision anterior shoulder reconstruction.. Am. J. Sports Med..

[4] (2005). Mazzocca, Brown FMJr, Carreira DS, Hayden J, Romeo AA. Arthroscopic anterior shoulder stabilization of collision and contact athetes.. Am. J. Sports Med..

[5] Brophy RH, Marx RG (2009). The treatment of traumatic anterior instability of the shoulder: nonoperative and surgical treatment.. J. Arthroscopy.

[6] Robinson C.M., Howes J., Murdoch H., Will E., Graham C. (2006). Functional outcome and risk of recurrent instability after primary traumatic anterior shoulder dislocation in young patients.. J. Bone Joint Surg. Am..

[7] Porcellini G., Campi F., Pegreffi F., Castagna A., Paladini P. (2009). Predisposing factors for recurrent shoulder dislocation after arthroscopic treatment.. J. Bone Joint Surg. Am..

[8] Beighton P., Horan F. (1969). Orthopaedic aspects of the Ehlers-Danlos syndrome.. J. Bone Joint Surg. Br..

[9] OBrien S.J., Pagnani M.J., Fealy S., McGlynn S.R., Wilson J.B. (1998). The active compression test: A new and effective test for diagnosing labral tears and acromioclavicular joint abnormality.. Am. J. Sports Med..

[10] Seoung-H K., Kwon-Ick H., Kye-Young H. (1999). Biceps load test: A clinical test for SLAP lesions in shoulders with recurrent anterior dislocations.. Am. J. Sports Med..

[11] Gagey O.J., Gagey N. (2001). The hyperabduction test.. J. Bone Joint Surg. Br..

[12] Jr B., Burkhart S.S., de Beer J.F. (2006). The bear.hug test for diagnosing a subscapularis tear.. Arthroscopy.

[13] Neviaser R.J., Neviaser T.J., Neviaser J.S. (1993). Anterior dislocation of the shoulder and rotator cuff rupture.. Clin. Orthop. Relat. Res..

[14] Pevny T., Hunter R.E. (1998). Freeman Jr. Primary traumatic shoulder dislocation in patients 40 years of age and older.. Arthroscopy.

[15] Toolamen G., Hildingsson C., Hedlung T., Knibestol M., Oberg L. Early complications after anterior dislocation of rhe shoulder in patients over 40 years.. Acta Orthop. Scand..

[16] Visser C.P., Coene L.N., Brand R., Tavy D.L. (1999). The incidence of nerve injury in anterior dislocation of the shoulder and its influence on functional recovery. A prospective clinical and EMG study.. J. Bone Joint Surg. Br..

[17] Itoi E., Lee S.B., Berglund L.J., Berge L.L., An K.N. (2000). The effect of a glenoid defect on anteroinferior stability of the shoulder after Bankart repair: a cadaveric study.. J. Bone Joint Surg. Am..

[18] Sugaya H., Moriishi J., Dohi M., Kon Y., Tsuchiya A. (2003). Glenoid rim morphology in recurrent anterior glenohumeral instability.. J. Bone Joint Surg. Am..

[19] Chen A.L., Hunt S.A., Hawkins R.J., Zuckerman J.D. (2005). Management of bone loss associated with recurrent anterior glenohumeral instability.. Am. J. Sports Med..

[20] Yamamoto N., Itoi E., Abe H., Minagawa H., Seki N., Shimada Y., Okada K. (2007). Contact between the glenoid and the humeral head in abduction, external rotation, and horizontal extension: a new concept of glenoid track.. J. Shoulder Elbow Surg..

[21] Randelli P., Ragone V., Carminati S., Cabitza P. (2012). Risk factors for recurrence after Bankart repair a systematic review.. Knee Surg. Sports Traumatol. Arthrosc..

[22] Pavlov H., Warren R.F., Weiss C.B., Dines D.M. (1985). The roentgenographic evaluation of anterior shoulder instability.. Clin. Orthop. Relat. Res..

[23] Huysmans P.E., Haen P.S., Kidd M., Dhert W.J., Willems J.W. (2006). The shape of the inferior part of the glenoid: a cadaveric study.. J. Shoulder Elbow Surg..

[24] Gerber C., Nyffeler R.W. (2002). Classification of glenohumeral joint instability.. Clin. Orthop. Relat. Res..

[25] Boileau P., Villalba M., Héry J.Y., Balg F., Ahrens P., Neyton L. (2006). Risk factors for recurrence of shoulder instability after arthroscopic Bankart repair.. J. Bone Joint Surg. Am..

[26] Probyn L.J., White L.M., Salonen D.C., Tomlinson G., Boynton E.L. (2007). Recurrent symptoms after shoulder instability repair: Direct MR arthrographic assessmentcorrelation with second-look surgical evaluation.. Radiology.

[27] Latarjet M. (1958). Technique de la butée coracóidienne pré-glénöidienne dans le traitement des luxations récidivantes de lépaule.. Lyon Chir..

[28] Marquardt B., Garmann S., Schulte T., Witt K., Steinbeck J., Pötzl W. (2007). Results and factors affecting outcome of revision surgery for shoulder instability.. J. Shoulder Elbow Surg..

[29] Cho N.S., Yi J.W., Lee B.G., Rhee Y.G. (2009). Revision open Bankart surgery after arthroscopic repair for traumatic anterior shoulder instability.. Am. J. Sports Med..

[30] Cole B.J., LInsalata J., Irrgang J., Warner J.J. (2000). Comparison of arthroscopic and open anterior shoulder stabilization. A two to six-year follow-up study.. J. Bone Joint Surg. Am..

[31] Hobby J., Griffin D., Dunbar M., Boileau P. (2007). Is arthroscopic surgery for stabilisation of chronic shoulder instability as effective as open surgery? A systematic review and meta-analysis of 62 studies including 3044 arthroscopic operations.. J. Bone Joint Surg. Br..

[32] Creighton R.A., Romeo A.A., Brown F.M., Hayden J.K., Verma N.N. (2007). Revision arthroscopic shoulder instability repair.. Arthroscopy.

[33] Kim S.H., Ha K.I., Kim Y.M. (2002). Arthroscopic revision Bankart repair: a prospective outcome study.. Arthroscopy.

[34] Levine W.N., Arroyo J.S., Pollock R.G., Flatow E.L., Bigliani L.U. (2000). Open revision stabilization surgery for recurrent anterior glenohumeral instability.. Am. J. Sports Med..

[35] Boone J.L., Arciero R.A. (2010). Management of failed instability surgery: how to get it right the next time.. Orthop. Clin. North Am..

[36] Cho N.S., Yi J.W., Lee B.G., Rhee Y.G. (2009). Revision open Bankart surgery after arthroscopic repair for traumatic anterior shoulder instability.. Am. J. Sports Med..

[37] Zabinski S.J., Callaway G.H., Cohen S., Warren R.F. (1999). Revision shoulder stabilization: 2- to 10-year results.. J. Shoulder Elbow Surg..

[38] Chen A.L., Hunt S.A., Hawkins R.J., Zuckerman J.D. (2005). Management of bone loss associated with recurrent anterior glenohumeral instability.. Am. J. Sports Med..

[39] Helfet A.J. (1958). Coracoid transplantation for recurring dislocation of the shoulder.. J. Bone Joint Surg. Br..

[40] Hovelius L., Sandström B., Saebö M. (2006). One hundred eighteen Bristow-Latarjet repairs for recurrent anterior dislocation of the shoulder prospectively followed for fifteen years: study II-the evolution of dislocation arthropathy.. J. Shoulder Elbow Surg..

[41] Schmid S.L., Farshad M., Catanzaro S., Gerber C. (2012). The Latarjet procedure for the treatment of recurrence of anterior instability of the shoulder after operative repair: a retrospective case series of forty-nine consecutive patients.. J. Bone Joint Surg. Am..

[42] Lafosse L., Lejeune E., Bouchard A., Kakuda C., Gobezie R., Kochhar T. (2007). The arthroscopic Latarjet procedure for the treatment of anterior shoulder instability.. Arthroscopy.

[43] Boileau P., Mercier N., Roussanne Y., Thélu C.É., Old J. (2010). Arthroscopic Bankart-Bristow-Latarjet procedure: the development and early results of a safe and reproducible technique.. Arthroscopy.

[44] Lafosse L1 (2010). Boyle S Arthroscopic Latarjet procedure.. J. Shoulder Elbow Surg..

[45] Warner J.J., Gill T.J., Ohollerhan J.D., Pathare N., Millett P.J. (2006). Anatomical glenoid reconstruction for recurrent anterior glenohumeral instability with glenoid deficiency using an autogenous tricortical iliac crest bone graft.. Am. J. Sports Med..

[46] Taverna E., DAmbrosi R., Perfetti C., Garavaglia G. (2014). Arthroscopic bone graft procedure for anterior inferior glenohumeral instability.. Arthrosc. Tech..

[47] Kraus N., Amphansap T., Gerhardt C., Scheibel M. (2014). Arthroscopic anatomic glenoid reconstruction using an autologous iliac crest bone grafting technique.. J. Shoulder Elbow Surg..

[48] Wolf E.M., Pollack M., Smalley C. (2007). Smalley C. Hill-Sachs “remplissage”. An arthroscopic solution for engaging Hill-Sachs lesion.. Arthroscopy.

[49] Wolf E.M., Arianjam A. (2014). Hill-Sachs remplissage, an arthroscopic solution for the engaging Hill-Sachs lesion: 2- to 10-year follow-up and incidence of recurrence.. J. Shoulder Elbow Surg..

[50] Yagishita K, Thomas BJ (2002). Use of allograft for large Hill-Sachs lesion associated with anterior glenohumeral dislocation a case report. Injury;.

[51] Gerber C, Lambert SM (1996). Allograft reconstruction of segmental defects of the humeral head for the treatment of chronic locked posterior dislocation of the shoulder.. J. Bone Joint Surgery.

[52] Martinez A.A., Calvo A., Domingo J., Cuenca J., Herrera A., Malillos M. (2008). Allograft reconstruction of segmental defects of the humeral head associated with posterior dislocations of the shoulder.. Injury.

[53] Miniaci A, Berlet G, Hand C, Lin A. (2008). A segmental humeral head allografts for recurrent anterior instability of the shoulder with large Hill-Sachs defects. A two to 8 years follow up J Bone Joint Surg Br.

